# Supplementary material for: Polarization and Thickness Dependent Absorption Properties of Black Phosphorus: New Saturable Absorber for Ultrafast Pulse Generation
Source: Sci Rep. 2015 Oct 30;5:15899. doi: 10.1038/srep15899 (PMC4626849; doi:10.1038/srep15899)
Supplement: Supplementary Information [file srep15899-s1.doc]

Supplementary Information

**Polarization and Thickness Dependent Absorption Properties of Black Phosphorus: New Saturable Absorber for Ultrafast Pulse Generation**

**Diao Li^1,2,+^, Henri Jussila^1,+^, Lasse Karvonen^1^, Guojun Ye^3,4^, Harri Lipsanen^1^, Xianhui Chen^3,4,5^, & Zhipei Sun^1,*^**

^1^ Department of Micro- and Nanosciences, Aalto University, Tietotie 3, FI-02150 Espoo, Finland

^2^ Institute of Photonics & Photo-Technology, Northwest University, Xi’an, 710069, China

^3^ Hefei National Laboratory for Physical Science at Microscale and Department of Physics, University of Science and Technology of China, Hefei, 230026, China

^4^ Key Laboratory of Strongly-coupled Quantum Matter Physics, University of Science and Technology of China, Chinese Academy of Sciences, Hefei, 230026, China

^5^ Collaborative Innovation Center of Advanced Microstructures, Nanjing 210093, China

^*^ [zhipei.sun@aalto.fi](mailto:zhipei.sun@aalto.fi)

^+^ These authors contributed equally to this work.

**Contents:**

**1. Atomic force microscopy images of black phosphorus flakes on the fiber ends**

**2. Polarization dependent Raman spectroscopy results**

**3. Linear absorption spectra at 1550 nm**

**4. Fluence dependent absorption measurement setup and nonlinearity fit**

**5. Output power of the Q-switched laser**

**6. Radio-frequency spectrum of the mode-locked laser**

**7. Output polarization state characterization of the pulsed lasers**

**8. References**

1. Atomic force microscopy images of black phosphorus flakes on the fiber ends

Atomic force microscopy (AFM) was used to study the thickness of transferred black phosphorus (BP) thin films on fiber ends. Supplementary Figure 1 shows two examples (~1100 nm and ~320 nm) of AFM images taken from the transferred BP flakes and the line profiles along the blue lines. The dashed white lines schematically show the location of the fiber cladding edge, and the green circle indicates the fiber core.

**Supplementary Figure 1:** AFM images of two BP flakes transferred on the fiber ends and the line profile along the blue line. **a.** and **c.** for a ~1100 nm thick BP film; **b.** and **d.** for a ~320 nm thick BP film.

**2. Polarization dependent Raman spectroscopy results**

The Raman spectra were carried out with a confocal Raman microscope (Witec alpha 300R) equipped with a frequency doubled Nd:YAG green laser (λ = 532 nm). We also carried out the polarization dependent Raman spectrum. The intensity of three peaks (located at the wavenumbers of 363 cm^-1^, 441 cm^-1^ and 469 cm^-1^), associated to A_g_^1^, B_2g_ and A_g_^2^ vibration modes in BP crystal lattice, are plotted with the different analyser angles, and shown in Supplementary Figure 2. Similar anisotropic property has also been observed with other groups, providing a unique method for determining the BP crystalline direction.


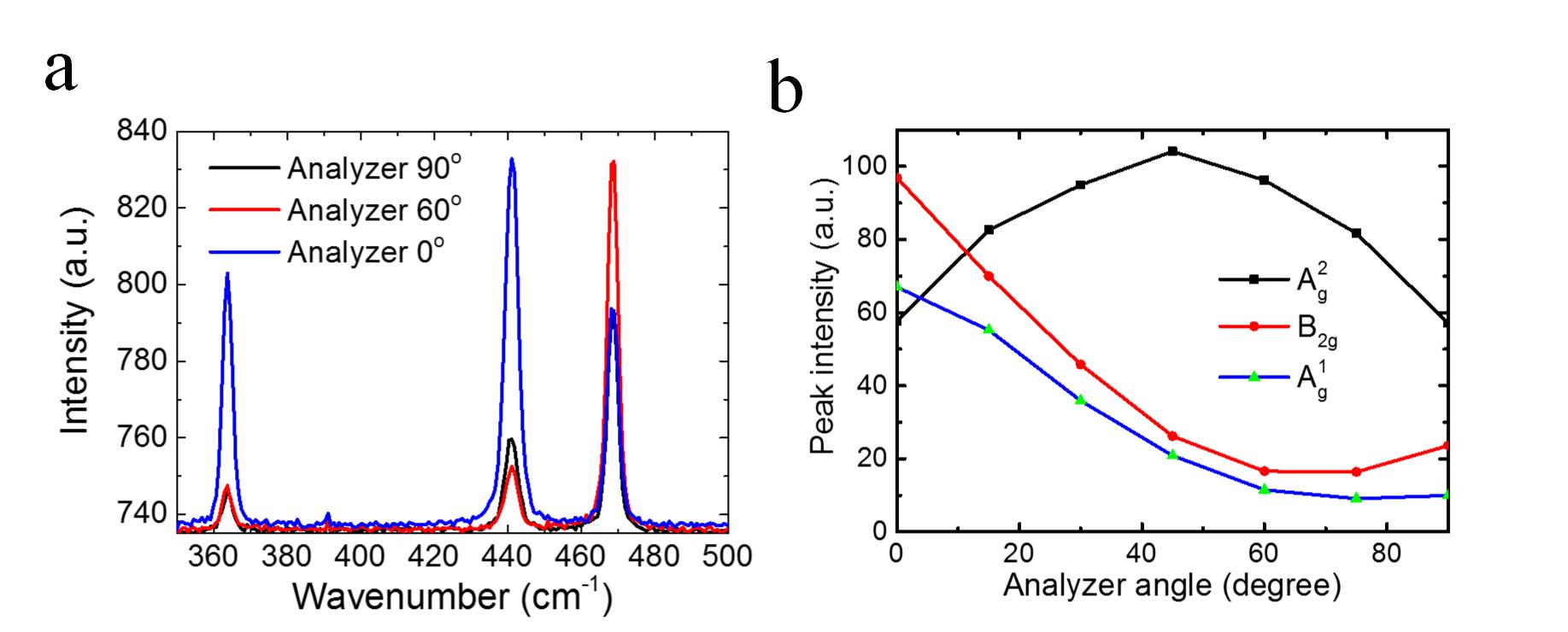


**Supplementary Figure 2:** Polarization dependent Raman intensity of three peaks (located at the wavenumber of 363 cm^-1^, 441 cm^-1^ and 469 cm^-1^), associated to A_g_^1^, B_2g_ and A_g_^2^ vibration modes in BP crystal lattice.

**3. Linear absorption spectra at 1550 nm**

Supplementary Figure 3 shows the linear absorption spectra at 1550 nm of thin films with different thicknesses. Transmission decreases with the increase of film thickness. The input light source for linear absorption is non-polarized.

**Supplementary Figure 3:** Linear absorption spectra at 1550 nm of different BP films.

**4. Fluence dependent absorption measurement setup and nonlinearity fit**

Schematic of the setup used in the fluence (F) dependent absorption measurements is shown in Supplementary Fig. 4 ^1^: A power-amplified home-made ultrafast fiber laser (~15 mW, 530 fs, 62 MHz) was employed to measure the saturable absorption property of the BP based fiber devices. A polarization controller was used to change the light polarization direction to measure polarization dependent saturable absorption performance. A double channel power meter (Ophir, Laserstar) was used to achieve high-accuracy measurement.


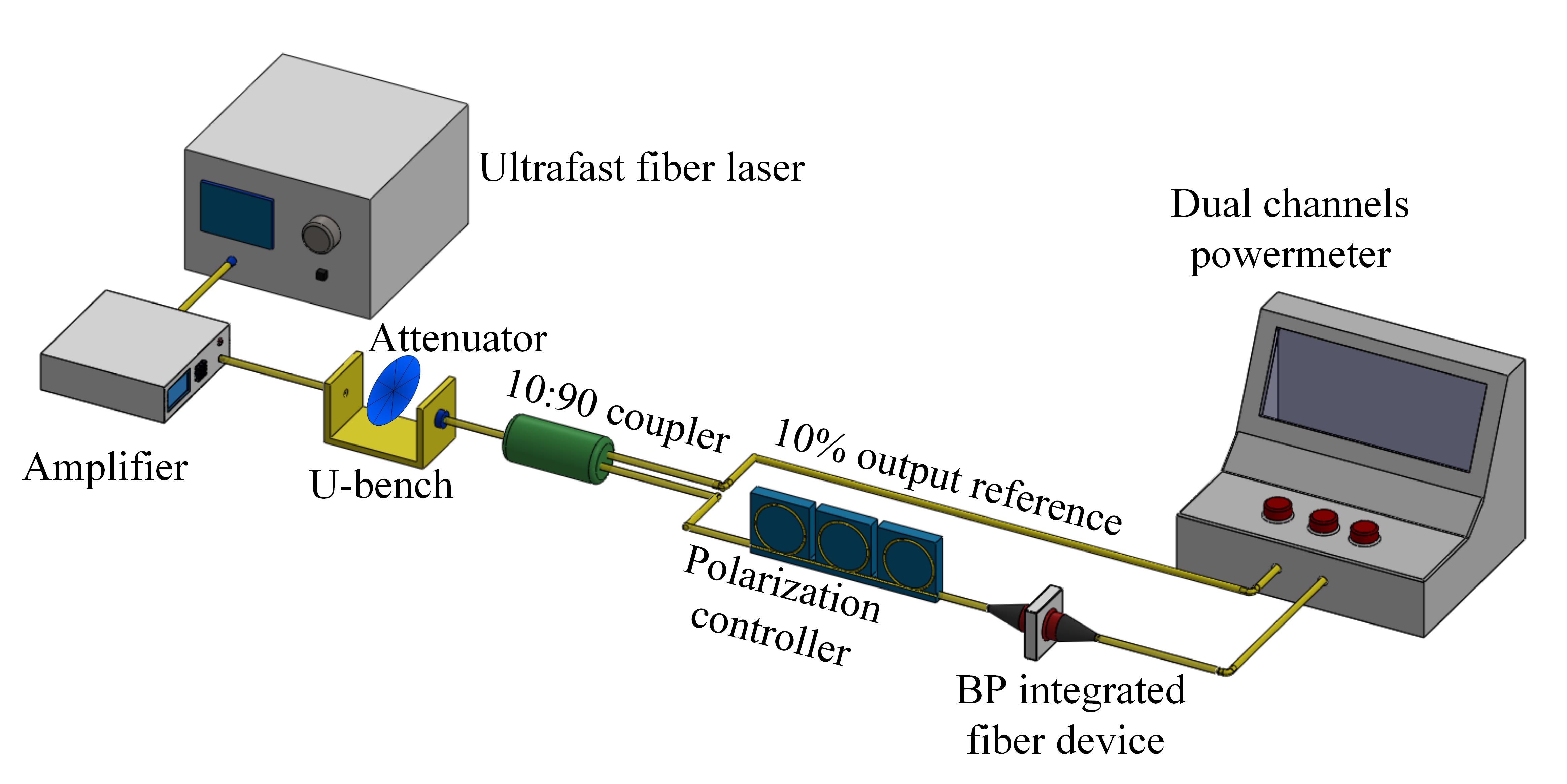


**Supplementary Figure 4:** Schematic of the setup used for nonlinear absorption measurement.

To estimate the saturation fluence and modulation depth from the nonlinear absorption curves, we use the following simplified formula to fit the measurement results ^2-3^


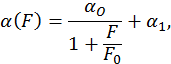


where
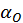
, and
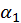
 are the absorption coefficients related to the power dependent absorption modulation and linear absorption, respectively.
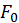
 is the saturation fluence.

**5. Output power of the Q-switched laser**

The typical output power as a function of pump power is given in Supplementary Fig. 5. The laser threshold pump power is ~11 mW. The output power increases to ~700 µW, when the pump power is 55 mW. The slope efficiency is ~1.5%, which is typical for such Erbium-doped fiber lasers.

**Supplementary Figure 5:** Typical output power of our laser as a function of the pump power.

**6. Radio-frequency spectrum of the mode-locked laser**

The radio-frequency spectrum of our BP mode-locked fiber laser is given in Supplementary Fig. 6.

**Supplementary Figure 6:** Radio frequency spectrum measured up to 500 MHz. The resolution bandwidth is 3 MHz.

**7. Output polarization state characterization of the pulsed lasers**

The schematic setup used for output polarization state characterization of the lasers is shown in Supplementary Fig. 7. A rotatable optical polarizer was used to extract the polarized vector in 360-degree. A power meter (Ophir, Laserstar) was used to measure the intensity of the transmitted light after the polarizer, as a function of ϕ (shown in Supplementary Fig.7).

**Supplementary Figure 7:** Schematic setup for laser output polarization state characterization.

**8. References**

1. Hasan, T.; Sun, Z.; Wang, F.; Bonaccorso, F.; Tan, P. H.; Rozhin, A. G.; Ferrari, A. C. Nanotube-Polymer Composites for Ultrafast Photonics. *Adv. Mater.* **21**, 3874, (2009).

2. Keller, U. Ultrafast solid-state lasers. Elsevier, Amsterdam, (2004).

3. Garmire, E. Resonant optical nonlinearities in semiconductors. *IEEE J. Sel. Top. Quantum Electron.* **6**, 1094, (2000).
